# Setting the stage: the sleek cell-cycle machinery of the liverwort *Marchantia polymorpha*

**DOI:** 10.1093/plcell/koag111

**Published:** 2026-04-16

**Authors:** Julie Robinson

**Affiliations:** Assistant Features Editor, The Plant Cell, American Society of Plant Biologists; HudsonAlpha Institute for Biotechnology, Huntsville, AL 35806, United States

Just like the army of stagehands behind your favorite stage production, the cell cycle is a tightly controlled system that proceeds according to a very specific program. While its purpose is conserved—making sure the show goes on by organizing cell growth and division and ensuring the faithful preservation of genetic material between generations—the number of players and their functions vary between different organisms.

The cell cycle is the backdrop against which all of a cell's inner workings occur. Despite its model system status, *Arabidopsis thaliana* contains so many duplicates of cell-cycle proteins that it is difficult to discern their highly specialized and often redundant functions (Lee et al. 2024; Vukasinovic et al. 2025). To address this challenge, **Romani, Bonter, and colleagues (Romani et al. 2026)** performed a phylogenetic analysis of cell-cycle genes in 27 diverse eukaryotic species that revealed a much more condensed set of such genes in the liverwort *Marchantia polymorpha* than in flowering plants, and proceeded to further characterize these genes. The potential resemblance between the simplified set of cell-cycle genes in Marchantia and that of the common ancestor of land plants positions Marchantia as an attractive model for studies of cell-cycle regulation in plants.

The pairing of certain cyclins with cyclin-dependent kinases (CDKs) directs cell-cycle progression. CDKA is conserved across eukaryotes, and CDKB is conserved across all green plants. Romani, Bonter, and colleagues analyzed previously published single-cell RNA-seq data from the vegetative gametophyte of Marchantia (Wang et al. 2023), then followed up with fluorescent imaging of translational reporters and generation of both overexpression and CRISPR-Cas9 knockout lines to characterize the function of Marchantia's cell-cycle machinery. They identified three subclusters of cell-cycle genes: one corresponding to G1-phase that included Mp*CYCD*;1, one to S-phase that included Mp*CYCA*, and one to G2/M-phase that included Mp*CYCB*;1 and Mp*CDKB*.

When a cell proceeds through the G1/S transition, it commits to undergoing cell division (Rossi and Varotto 2002). Dormant Marchantia spores start dividing 29–48 h after light exposure (Attrill et al. 2024). The authors report high Mp*CYCD;1* transcript levels 24 h after light exposure, consistent with a role for this cyclin in G1-phase and cell-cycle re-entry. Furthermore, MpCYCD;1 protein was found to accumulate in the nucleus during G1-phase and then quickly degrade before prophase. Overexpression of Mp*CYCD;1* resulted in an increase in cellular division, leading to the formation of many small, undifferentiated cells. Indeed, expression of Mp*CYCD;1* under a promoter derived from differentiating rhizoids also resulted in an increase in cellular division that notably suppressed rhizoid development. Altogether, this evidence supports the classification of MpCYCD;1 as a D-type cyclin that regulates the G1/S transition in Marchantia.

The MpCYCA reporter accumulated in the nucleus during S-phase, and its degradation was accompanied by peak nuclear MpCYCB;1 levels during prophase. Overexpression of either B-type cyclin, Mp*CYCB;1* or Mp*CYCB;2*, yielded smaller plants as a result of reduced growth. Cells highly overexpressing Mp*CYCB;1* became arrested in mitosis, which is consistent with the degradation of B-type cyclins being necessary for mitotic exit (Murray et al. 1989). Finally, Romani, Bonter, and colleagues generated an Mp*KRP* (Kip-related protein) overexpression line that displayed large cells that had failed to undergo cell division, supporting a conserved role for KRP in plants.

More cell-cycle genes exist in Marchantia than have been discussed here, but, even still, they comprise a relative skeleton crew that can easily be used as phase-specific markers (Fig. 1), setting the stage for simplified cell-cycle research in plants that may have broader implications for our understanding of the cell cycle in all eukaryotes. Future research in Marchantia could characterize the cell-cycle machinery in the sporophyte stage and during meiosis.

**Figure 1:**
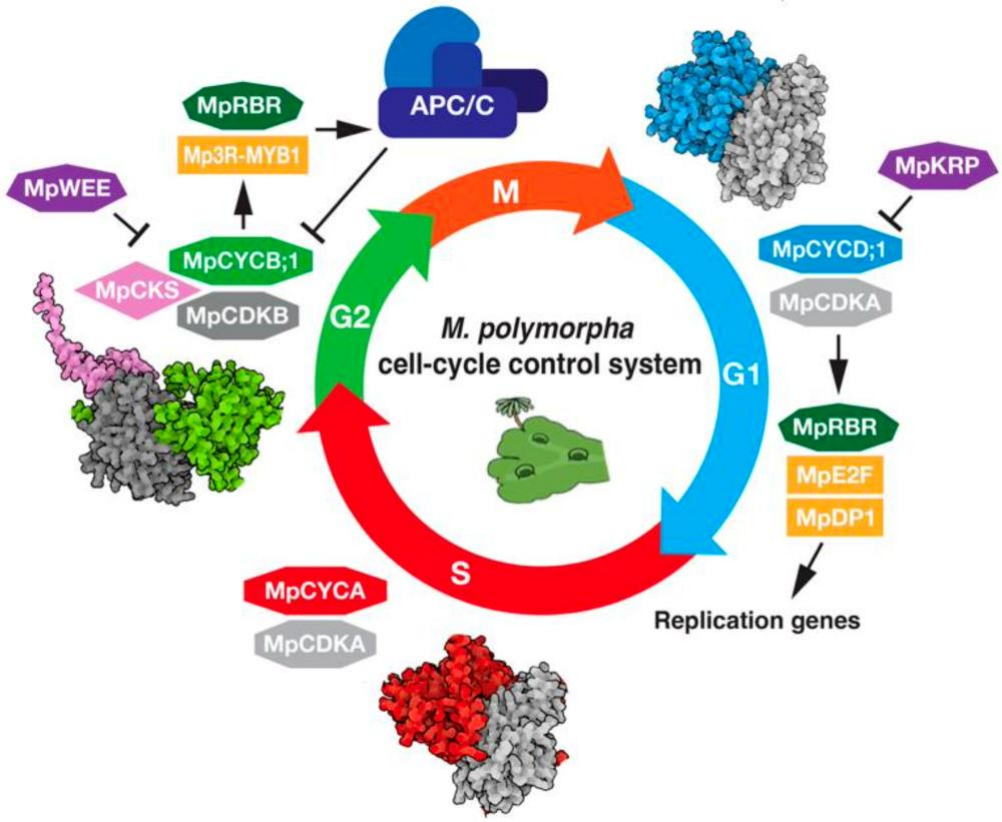
Key cell-cycle players in *Marchantia polymorpha*, as elucidated experimentally by Romani, Bonter, and colleagues as well as inferred from other model systems. Adapted from Romani et al. (2026), Figure 7.

## Recent related articles in *The Plant Cell*:

- Xu et al. (2025) characterized the role of APC/C coactivators in AUR1 regulation in Arabidopsis.
- Kim et al. (2024) described a role for DYRKP1 kinase in Chlamydomonas cell wall degradation.
- Ren et al. (2024) investigated the role of BNB-GLID in male germline fate determination in Marchantia.

## Data Availability

No new data were generated or analyzed in support of this article.
